# The description and number of undiscovered mammal species

**DOI:** 10.1002/ece3.3724

**Published:** 2018-03-04

**Authors:** Molly A. Fisher, John E. Vinson, John L. Gittleman, John M. Drake

**Affiliations:** ^1^ Odum School of Ecology University of Georgia Athens GA USA

**Keywords:** biodiversity, conservation, taxonomic effort, total number of species, unknown species

## Abstract

Global species counts are a key measure of biodiversity and associated metrics of conservation. It is both scientifically and practically important to know how many species exist, how many undescribed species remain, and where they are found. We modify a model for the number of undescribed species using species description data and incorporating taxonomic information. We assume a Poisson distribution for the number of species described in an interval and use maximum likelihood to estimate parameter values of an unknown intensity function. To test the model's performance, we performed a simulation study comparing our method to a previous model under conditions qualitatively similar to those related to mammal species description over the last two centuries. Because our model more accurately estimates the total number of species, we predict that 5% of mammals remain undescribed. We applied our model to determine the biogeographic realms which hold these undescribed species.

## 1 INTRODUCTION

The routine description of biological species not previously known to science shows clearly that the project to catalog life on earth may be only two‐thirds complete (Costello, Wilson, & Houlding, 2012; Pimm et al., 2014). With species extinction rates similar to description rates, it is exceedingly important to know how many species remain to be described (Costello, May, & Stork, 2013; Tedesco et al., 2014). Limited sampling of the world's biodiversity makes it impossible to directly count the total number of species that exist on Earth (Mora, Tittensor, Adl, Simpson, & Worm, 2011). Because many undescribed species may go extinct before they are known to science, ecological and conservation science must rely on statistical estimates of the total number of extant species (Costello et al., 2013; Tedesco et al., 2014). Experts disagree, however, about how to accurately determine the number of global species, with differing opinions on which analyses to run, the spatial distributions to analyze, what data quality is necessary, and even how to define a species (Mora et al., 2011). These varied approaches lead to estimates ranging sixfold, from ~2 million to ~13 million for the total number of species (Costello et al., 2012; Scheffers, Joppa, Pimm, & Laurance, 2012).

Rather than modeling how many species remain to be described, some researchers have used species descriptions since the last checklist (Hoffmann et al., 1993; Wilson & Reeder, 2005) to analyze the completeness of species lists and other patterns of discovery (Ceballos & Ehrlich, 2009; Patterson, 2000). Although these analyses do not provide estimates of how many species remain undescribed, these studies suggest that more species do remain, and probably more than had previously been expected (Ceballos & Ehrlich, 2009; Patterson, 2000). Others have tried to actually estimate the total number of species remaining to be described, both regionally and globally. Essl, Rabitsch, Dullinger, Moser, and Milasowszky (2013) predicted that 0.4%–3% of existing wide‐ranging European faunal species and 5%–19% of European endemics remain undescribed. These numbers indicate that, even in a well‐known region, there remain many undiscovered species (Essl et al., 2013). Tedesco et al. (2014) estimated that about 300 mammal species remain undescribed, along with about 3,000 freshwater fish and 100 freshwater bivalves. These estimates were used to determine how many of those undescribed species are already extinct (Tedesco et al., 2014). But the common denominator for all of these studies is using species description data to analyze the completeness of species lists and determine just how much biodiversity remains unknown.

Mora et al. (2011) described three approaches to estimating the number of species: macroecological patterns, diversity ratios, and taxonomic patterns. Of these, taxonomic patterns appear to provide the most consistently reliable estimates of species richness (Mora et al., 2011), typically involving models of species accumulation curves with extrapolation. Assuming that the description rate of new species declines with time, species accumulation models estimate species richness from description data (Costello & Wilson, 2011; Mora, Tittensor, & Myers, 2008; Wilson & Costello, 2005). Species accumulation modeling approaches provide the most accurate estimates of the total number of species when accumulation curves approach asymptotic levels (Mora et al., 2011). This suggests that to estimate the total number of species when accumulation curves are not obviously asymptotic, models should include other contributions to species description events.

Joppa, Roberts, and Pimm (2011) observed that the number of taxonomists publishing species descriptions correlated with the number of species described in every 5‐year period, and proposed a model incorporating *taxonomic effort*, defined as the number of taxonomists who published species descriptions in a time interval. The number of taxonomists working in each year increased over time, with a correlated increase in the number of new species described in each year, which led to the definition of a term for *taxonomic efficiency*, which is the number of species described per unit effort (Joppa, Roberts, Myers, & Pimm, 2011; Joppa, Roberts, & Pimm, 2011; Pimm, Jenkins, Joppa, Roberts, & Russell, 2010). For Joppa, Roberts, and Pimm (2011) and Joppa, Roberts, Myers, et al. (2011), including taxonomic effort and taxonomic efficiency increased the accuracy of estimates obtained using species accumulation models.

This model has been used to estimate the total number of plants, amphibians, and mammals both globally and regionally with birds only estimated regionally (Giam et al., 2012; Joppa, Roberts, & Pimm, 2011; Joppa, Roberts, Myers et al., 2011; Pimm et al., 2010). However, in general, mammals have been mostly ignored when making global species diversity estimates. Mammals are relatively rare, charismatic, and endangered, causing them to be of high importance for both economic and conservation concerns. The available information on mammal geographic distributions allows for analyses at both global and regional spatial scales. As a greatly imperiled taxonomic group, mammals are an intrinsically interesting group which provide a unique opportunity to test methods using a group that is almost complete and the least speciose, suggesting that any method that works with mammals is likely to work with other well‐known taxa. A previous attempt to estimate the total number of mammal species underestimated global land mammal species using a previous iteration of the Joppa, Roberts, and Pimm (2011) method (Giam et al., 2012). Additionally, when estimating regional species diversity, the previous method included species across multiple realms, resulting in inflated regional estimates (Giam et al., 2012). To address these inaccuracies, we modified a newer iteration of the Joppa, Roberts, Myers, et al. (2011) model to better estimate the total number of mammal species, attempting to predict how many and where undescribed species are yet to be found.

## 2 METHODS

### 2.1 Data

We constructed regional mammal description curves and global mammal description curves from *Mammal Species of the World* (Wilson & Reeder, 2005) and the International Union for Conservation of Nature and Natural Resources (IUCN) mammal data (IUCN 2015). Because Wilson and Reeder (2005) stop with species described in 2003, the Wilson and Reeder (2005) designation for species binomial was used for those species. For species described after 2003, the IUCN designation was used (IUCN 2015). All newly added IUCN species were checked for possible synonyms to previously described species, with any species that had been previously described as a synonym removed from the dataset. See Table S1 for list of included species. For model fitting, the number of species and taxonomists was collated for each 5‐year period from 1760 through 2010.

Previous attempts to estimate the total number of mammal species have used different methods of assigning a species description to a taxonomist (Giam et al., 2012; Joppa, Roberts, Myers et al., 2011). For instance, in Giam et al. (2012), it was assumed that a maximum of two taxonomists was responsible for the description of a species in which these two taxonomists were the first two corresponding authors of the description monograph. But, this method causes double counting of taxonomists due to the fact that each taxonomist was credited for the description of the species separately, resulting in an inflated estimate for the number of taxonomists working in an interval. In Joppa, Roberts, and Pimm (2011), the importance of taxonomist designation was investigated. Joppa, Roberts, and Pimm (2011) used three measures of taxonomic effort to see whether differing measures have any effect on the overall model. Their results suggest that the model's estimate is unaffected by the method of taxonomic assignment (Joppa, Roberts, Myers et al., 2011). We used the entire authorship of the citation for the description paper as the taxonomists for the description of the species. For example, if the authorship of two citations is “Mares & Braun” and “Mares, Braun, Barquez & Diaz” then these would be counted as distinct “taxonomists” for our purposes. Although our method of counting taxonomists could also be considered to be counting manuscripts rather than taxonomists, the count reflects an increased number of taxonomists working in more recent times as each published manuscript is more differentiated. This differentiation is a result of differing author orders in publications recently resulting in each order receiving a single count, whereas when there were only one or two authors, multiple manuscripts were counted as a single taxonomist working during that period.

**Figure 1:**
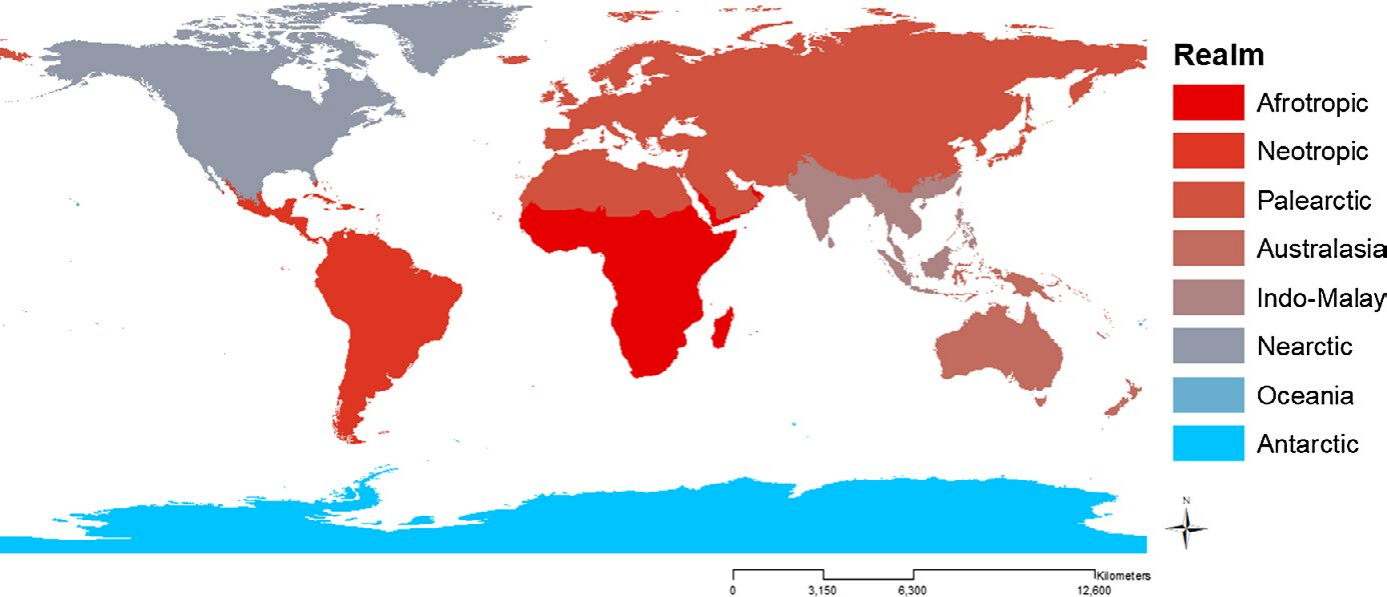
Map of Biogeographic Realms. Cooler (blue) colors represent regions with fewer undiscovered species, and warmer (red) colors represent regions with more undiscovered species

To investigate geographic variation in undescribed species, we constructed region‐specific description curves by binning species based upon geographic range (Figure 1). We created a model in ArcGIS 10.0 (Esri, 2011) to determine which region a species' geographic range fell within for the majority of the range (see Figure S1 in the Supporting Information; IUCN 2015).

### 2.2 Model

To represent species removal from an unknown total species pool, we developed a model that represents species description as a relationship among total undescribed species, taxonomic efficiency (how effective taxonomists are at finding new species), and taxonomic effort (the number of taxonomists describing species; Joppa, Roberts, & Pimm, 2011; Joppa, Roberts, Myers et al., 2011). Motivated by Joppa, Roberts, and Pimm (2011), we assume that number of species described in a time interval (*S*_*i*_) is proportional to the number of taxonomists working (*T*_*i*_) in that interval and the unknown number of species remaining to be described (*S*_*U*_), via a coefficient that represents the taxonomic efficiency (*E*_*i*_):

(1) $$S_{i}\propto T_{i}\times S_{U}\times E_{i}.$$

Denoting the unknown total number of species by *S*_*T*_ and the number described up to time *i* by *D*_*i*_ *,* we have *S*_*U*_ **= *S*_*T*_–*D*_*i*_, which may be substituted into equation (1) to give

(2) $$S_{i}\propto T_{i}\times \left(S_{T}-D_{i}\right)\times E_{i},$$

the number described in interval *i* in terms of the total species pool and unknown coefficients. (See Table 1 for a list of parameters.) Our model is made more realistic by allowing taxonomic efficiency to vary over time, but only such that it strictly increases or remains constant, as scientific technique improves and knowledge accumulates. That is, we assume taxonomists only ever become more effective at describing new species as time progresses. A flexible parametric expression for taxonomic efficiency is the exponential function *ae* ^*bYi*^, where *Y*_*i*_ denotes the time interval bin (i.e., 0, 5, 10, … where 0 refers to the time bin from 1755 to 1760 and five refers to the bin from 1760 to 1765) and *a *>* *0 and *b *≥* *0 are estimated coefficients. This contrasts with Joppa, Roberts, and Pimm, (2011) and Joppa, Roberts, Myers, et al. (2011), who used a linear model (i.e., *a *+ *bY*_*i*_) on the grounds that no more complicated model was warranted by the data. Our model is equally complex (two unknown parameters) and has the added virtues of being everywhere nonnegative and approximately linear over short time periods but allowing for more curvature over longer time periods. Substituting this expression into equation (2), substituting *S*_*i*_ with *S*_*iest*_, the estimated number of species described in each 5‐year interval, and replacing the proportionality with equality, our model becomes

(3) $$S_{\mathrm{iest}}=T_{i}\times \left(S_{T}-D_{i}\right)\times ae^{bY_{i}},$$

**Table 1:** Results of applying models to full mammal data set, *S*_*T*_ is the total number of species, *a* is the intercept of the taxonomic efficiency function, *b* is the slope of the taxonomic efficiency function, and *z* is the scaling coefficient in the Gaussian maximizing function

	Starting Gaussian Linear	Gaussian Lower confidence interval	Estimated Gaussian Linear	Gaussian Upper confidence interval	Starting Poisson Exponential	Poisson Lower confidence interval	Estimated Poisson Exponential	Poisson Upper confidence interval
*S*_*T*_	5,970	5,556	5,558	5,560	5,970	5,792	5,860	5,928
*a*	8e−04	0.0056	0.0322	0.1837	8e−04	0.0007	0.0007	0.0008
*b*	2e−03	5e−06	1.7e−05	5.7e−05	0.002	0.0048	0.0054	0.0061
*z*	5	24.54	61.19	152.59	–	–	–	–

**Figure 2:**
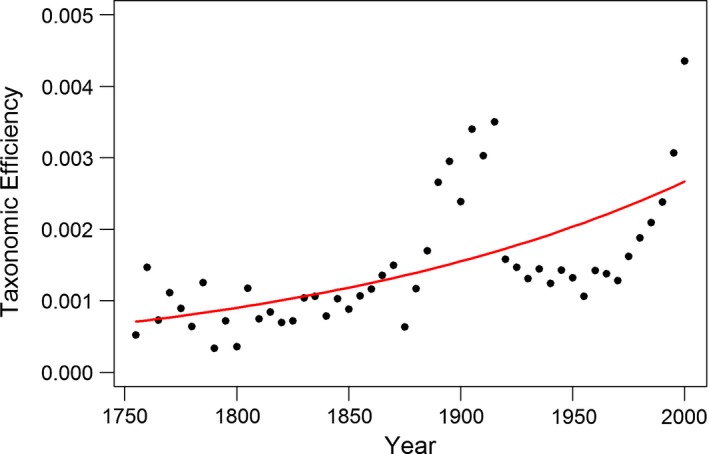
Taxonomic efficiency. Investigating the fit of the modeled efficiency function (red line) to the calculated efficiency for mammals (black dots). The points which do not follow the modeled efficiency function from 1890 to 1915 result from the ratio of species to taxonomists in those years. As seen in Figure 4, the number of species exceeds the number of taxonomists in a different pattern than expected

To investigate the fit of the taxonomic efficiency submodel, it is useful to observe the fit of the parameterized function *E*_*i*_ to the *calculated efficiency*, $CE_{i}=\left(\frac{S_{i}}{\left[S_{T}-D_{i}\right]T_{i}}\right)$ (See Figure 2). By viewing the fit of the parameterized function to the calculated efficiency, we observe how well the parameterization fits. A high correlation between the predicted taxonomic efficiency and the calculated efficiency would suggest that our function is working properly.

### 2.3 Maximum likelihood estimation

Maximum likelihood was used to estimate all model parameters, including *S*_*T*_ *,* total number of species (Bolker, 2008). From the Poisson assumption, the likelihood is given by,

$$L\left(a,b,S_{T}|S_{i}\right)=-\sum_{i=1}^{B}ln\left(e^{-ae^{\mathrm{bYi}}\times T_{i}\times \left(S_{T}-D_{i}\right)}\frac{{\left(ae^{\mathrm{bYi}}\times T_{i}\times \left(S_{T}-D_{i}\right)\right)}^{S_{i}}}{S_{i}!}\right),$$

where *B* refers to the number of time interval bins.

Maximum likelihood estimates for *a*,* b*, and *S*_*T*_ were obtained using the *optim* function in R v 3.01, utilizing the Nelder–Mead method for its robustness (Peressini, Sullivan, & Uhl, 1988; R Core Team 2015). To constrain *a* and *b* to be positive, we fitted the model in log coordinates for these variables. Optimization was repeated until the changes in estimates were reduced to less than 9 × 10^−6^. Parameter uncertainty was quantified using Wald's confidence intervals (Wald, 1940).

### 2.4 Simulation study

To investigate the robustness of our model, differing from Joppa, Roberts, Myers, et al. (2011) both in the assumption of a Poisson data distribution and the exponential model of taxonomic efficiency, we performed a simulation study to compare the estimation from our model to the estimation of Joppa, Roberts, Myers, et al. (2011). The historical process of species description resembles the likelihood that molecules in a vessel will collide and react with each other. To simulate the Markov Chain Monte Carlo process of species description, data were simulated using Gillespie's Direct Method (Gillespie, 1976) where each *event* was the description of a new species. Time between each event, a value determined by Gillespie's Direct Method, is drawn from an exponential distribution determined by the mammal species description curve.

To assess the models in varying situations, four scenarios were explored. The first scenario is the simplest, and each scenario after either adds a parameter or noise to the simulation to increase complexity and more closely resemble actual species description. In scenarios 1 and 2, true taxonomic efficiency was given by the model‐assumed exponential function $E=ae^{bY_{i}}$. Scenario 1 was the simplest scenario where taxonomic efficiency was constant without noise (*a *≈* *0.001, *b *=* *0). Set 2 allowed for the taxonomic efficiency to increase exponentially without noise (*a *=* *0.001, *b *=* *0.005). Scenarios 3 and 4 introduced noise in taxonomic efficiency with $E∼\Gamma (ae^{bY_{i}},1)$, where Γ denotes the gamma distribution. Simulation set 3 held the shape parameter constant (*a *≈* *0.001, *b *=* *0), and simulation set 4 allowed for an exponential increase in taxonomic efficiency (*a *≈* *0.001, *b *=* *0.005). For each simulation set, the total number of mammal species (*S*_*T*_) was held constant at 5,860 species and the number of taxonomists describing species in each 5‐year interval was taken from the observed data.

To analyze the performance of each method, we calculated the average total number of species and coverage, the proportion of the simulations for which the confidence interval of the resulting estimation of *S*_*T*_ included the actual value of *S*_*T*_, for each method in each experiment.

### 2.5 Geographic realm identification

To assign each described species to a biogeographic realm (The Nature Conservancy 2002), the geographic range of each mammal species, as published by the IUCN (IUCN 2015), was analyzed in ArcGIS v 10.1 (Esri, 2011). An ArcGIS tool was created which iterated through each species, calculating how much of each species' geographic range occupied each biogeographic realm (Figure 1; Esri, 2011). The realm that contained the greatest portion of a species' range was assigned as the realm for that species (Table S2). Only one species had a geographic range with less than 50% of its range within a single realm and only 4.29% of species have between 50% and 75% in a single realm, leaving 95.69% of species with more than 75% of species ranges found in a single biogeographic realm. The final assignments of species to biogeographic realms resulted in each species being assigned to a single realm, therefore, preventing double counting of species across realms. If a species were counted in each biogeographic realm in which it appears, there would be an artificial inflation in the total number of species. Once each species was assigned to a biogeographic realm, we then applied our model to each realm separately (See Table 2).

**Table 2:** Total number of mammal species by biogeographic realm

	Known	*S*_*T*_ lower bound	*S*_*T*_ upper bound	*S*_*T*_	Unknown	Percent unknown
Afrotropics	1,195	1,252	1,383	1,317	122	9.3
Australasia	678	699	778	739	61	8.3
Indo‐Malay	822	834	865	849	27	3.2
Nearctic	391	390	402	396	5	1.3
Neotropics	1,455	1,510	1,583	1,546	91	5.9
Palearctic	720	725	869	797	77	9.7

## 3 RESULTS

### 3.1 Simulation Study

**Figure 3:**
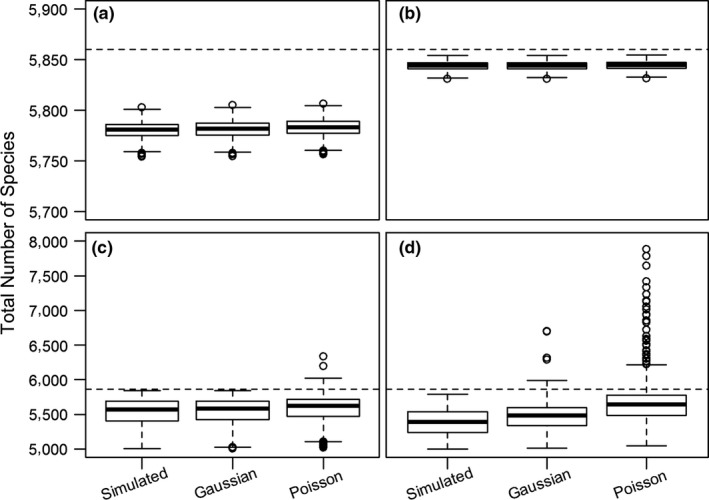
Boxplots of simulation results, with the middle line showing the median. Simulated boxes are the number of species simulated to have been described. Gaussian boxes refer to the estimated total number of species using the Joppa et al. method. Poisson boxes refer to the estimated total number of species using our method. a–d refer to scenarios 1‐4, respectively. Dashed lines refer to the actual total number of species

**Figure 4:**
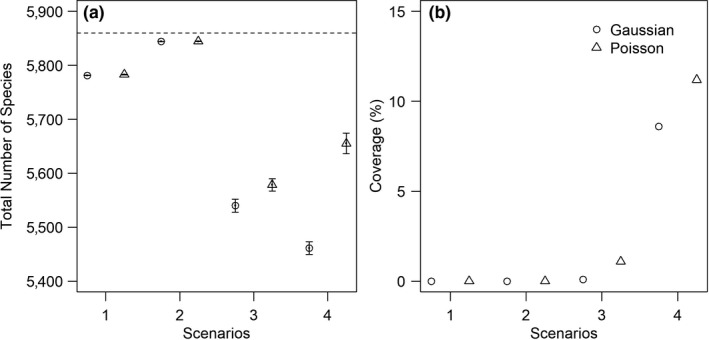
Simulation study results. Panel (a) shows the model bias and total number of species averages from simulated data. Dashed line shows true value. Error bars show 95% confidence interval of the average distance from the true number of species. Panel (b) shows statistical coverages (percent of estimates including the true value) from simulated data

Figures 3 and 4 summarize the results of our simulation study. In simulations, our method provided estimates closer to the actual number of species than the Joppa et al. method (Figures 3 and 4a). For scenarios 1 and 2, the estimates show low variance and low bias, resulting in estimates that are close to the true value for the total number of species on average and with a very small confidence interval (Figure 3a and b). For experiments 3 and 4, the estimates show higher variance and bias, resulting in estimates that are farther from the true value for the total number of species and with a larger confidence interval (Figure 3c and d). Coverage was larger for our method than for the Joppa et al. method (Figure 4b), although still very low. Because the confidence intervals were small and rarely included the actual value, the coverage was small for all simulations.

### 3.2 Estimated global number of mammal species

**Figure 5:**
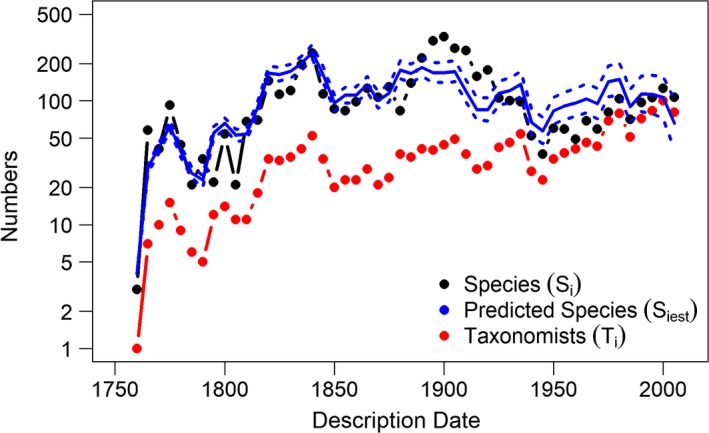
Mammal species discovered per 5‐year interval on a log scale. The dashed blue lines represent the confidence interval around the estimated number of species in each 5‐year interval (*S*_*iest*_)

Our model predicts the total number of species that currently exist. The plot of time against number of species shows that our model fits the actual number of species described per 5‐year period, with the Pearson's correlation coefficient of 0.72 (Figure 5). We predicted 5860 (95% prediction interval: ±68) mammal species exist, suggesting that 303 (95% prediction interval: ±68) species remain to be described (Table 1), which is larger than the estimate using the Joppa, Roberts, Myers, et al. (2011) method by 5.16% or 302 species (Table 1).

### 3.3 Where to find the new species

Applied to description curves by biogeographic realm, our model suggests that the Afrotropics and Neotropics contain both the greatest number of mammal species and the most undescribed species (Table 2, Figure S2). All other realms are estimated to contain less than 100 unknown species (Table 2). In contrast, the Palearctic contains the greatest percent of unknown species (9.7%; Table 2).

## 4 DISCUSSION

To estimate the total number of mammal species that exist, we modified a species accumulation model that incorporates both taxonomic effort and taxonomic efficiency. We performed a simulation study to test the performance of our model, which we then applied to data on the global sequence of mammal descriptions. Finally, we applied our model to regional mammal description data to determine where we will find undescribed mammal species.

Our model builds on a number of previously published methods. We proposed a more realistic method of maximum likelihood estimation: using the Poisson distribution, rather than estimating a Poisson distribution through a Gaussian approximation. Of the species accumulation models, those that use a Poisson distribution for the distribution of species descriptions are the most consistent for data acquired from a sampling process with continuous intensity (Wilson & Costello, 2005). In contrast to the Gaussian assumption of Joppa, Roberts, and Pimm (2011), a Poisson reporting process allows for the variance to differ according to the mean rate of species description. Additionally, we used a more flexible model to approximate the taxonomic efficiency which uses an exponential function because it never goes negative, rather than forcing unrealistic conditions on a linear function. We then tested our model by performing a simulation study, which compares our model to the Joppa et al. model. Our simulations were studied under four scenarios, each one more complex and realistic. The differences between the results from each method in the first two scenarios (see Figure 4) are not nominally large, whereas in the last two scenarios, the estimates are more different from each other. The first two scenarios do not incorporate process error when simulating the data, allowing smoother simulated accumulation curves to be generated. Scenarios 3 and 4 assume a baseline gamma‐distributed process error, providing complex accumulation curves. The results of our simulation study suggest that our method is better able to handle more complex collection data. Although our model performs best, it suffers from low coverage and is biased toward low estimates. Although all estimates are different from the actual total, across all scenarios, our method consistently provides estimates closer to the total (Figure 4) and more often includes the actual value in its confidence intervals (Figure 5).

The simulation study justifies the application of this model on the more complex, real‐world mammal data. Our results suggest that there are a total of 5,860 mammal species, meaning that we have about 300 mammal species, or 5% of species left to be described. One interesting phenomenon that our data bring to light is an effect caused by world wars. The number of species described, the number of taxonomists working, and our measure of taxonomic efficiency show a decline in response to both World War I (1914–1918) and World War II (1939–1945). This decline appears in the real data and is matched by the estimates of our model. As the ability for taxonomists to travel the world is often impaired by the social and political conflicts occurring, our model is able to make predictions despite irregular, nonlinear description, and efficiency curves. This phenomenon also suggests that future global conflicts may have a significant impact on our speed and efficiency at finding new species.

The regional and global mammal species estimates are useful for conservationists to know how much effort and where to concentrate such efforts before the species disappear (Dobson, Lafferty, Kuris, Hechinger, & Jetz, 2008). To determine the number of mammal species currently found in each geographic realm, we assigned each mammal species to a biogeographic realm and then ran our model for each regional grouping of mammals. The realm we predict to have the greatest number of undescribed species is the Afrotropics (Table 2). These results underscore the important relationship between range distribution and taxonomic effort by demonstrating that the places where the most effort has been expended are places that have the fewest remaining species (Pimm et al., 2014). Most species remaining undescribed are found in tropical regions (Neotropic and Afrotropic realms), which is also home to the greatest concentration of biodiversity (Joppa, Roberts, Myers et al., 2011; Patterson, 2000). To the contrary, the highest percent of unknown species is predicted in the Palearctic (Table 2). Although this region is fairly well known and investigated, there are some places, like Siberia, which may yet hide mammalian biodiversity.

To test the robustness of our model, we can compare the global estimate of total mammal species with the sum of the *S*_*T*_ estimates for each region. When comparing our method to Giam et al. (2012), our summed regional *S*_*T*_ estimates were within 100–200 species of the estimate based on the complete mammal data set, whereas theirs was not. Our estimates are based on larger geographic areas than investigated in most previous studies to ensure a large enough data set to get accurate results. However, Tedesco et al. (2014) estimated a similar number of undescribed mammal species remaining as we do, both overall and within Australasia. Even so, the limited number of mammal species found in some realms (Oceania and Marine realms) does not allow accurate estimates for the total number of species regionally, without compromising global estimates. Our results are much less biased as species were only designated to a single realm, eliminating the possibility of double counting. While keeping the estimates as unbiased as possible, we were able to predict where most undescribed mammal species will be found: tropical regions.

Our model predicts the total number of species that exist, based on taxonomic effort, taxonomic efficiency, and how many species have been described over time. Here, we applied our model to mammal species description curves, but the next step is to apply this model to other taxonomic groups. The original Joppa et al. method has been applied to multiple taxonomic groups, such as plants and the biota of Brazil (Pimm & Joppa, 2015; Pimm et al., 2010), suggesting that our model might perform similarly across taxonomic groups. It would also be interesting to investigate the description curves of mammalian orders, to determine how many of each order remain to be described. The biggest worry with disaggregating mammals into smaller taxonomic groups is that the quantity of data for each curve significantly decreases with each grouping. While our model predicts the number of species remaining to be described, it does not predict anything about those species such as their biology, evolution, extinction risk, or rarity. We have applied our model to geographic regions to help predict where these species will be found, but even those results are not specific. Further breakdown of regions, without double counting, is nearly impossible with our model due to minimum data requirements as well as the difficulty to place species into much smaller regions without requiring the method of species counting to be changed. Although our model was created to be applicable to more taxonomic groups based on the modified taxonomic efficiency function, a future study might look at a nonparametric approach.

## CONFLICT OF INTEREST

None declared.

## AUTHOR CONTRIBUTION

MF performed modeling work and analyzed output data. JV performed simulation study and assisted with modeling work. JD assisted with modeling work and idea development. JG and MF envisioned the project. MF wrote the first draft of the manuscript, and all authors contributed substantially to revisions.

## DATA ACCESSIBILITY

Should the manuscript be accepted, the data supporting the results will be archived in an appropriate public repository such as Dryad or Figshare and the data DOI will be included at the end of the article.
