# Supplementary material for: The description and number of undiscovered mammal species
Source: Ecol Evol. 2018 Mar 4;8(7):3628–35. doi: 10.1002/ece3.3724 (PMC5901171; doi:10.1002/ece3.3724)

# Supplement

Table S1: List of all Species included, with Date of Description, and Describing Taxonomist for each

Table S2: Percentage of Species Range in each Biogeographic Realm. Only one species has less than 50% of its range in any single realm and only 4.29% of species have between 50% and 75% in a single realm.

Figure S1: Model used to assign species to biogeographic realms using ArcGIS 10.0 (ESRI 2011). The blue circles represent input data, the orange hexagon is the iteration tool, the yellow rounded rectangles are ArcGIS tools, and the green circles are output data.


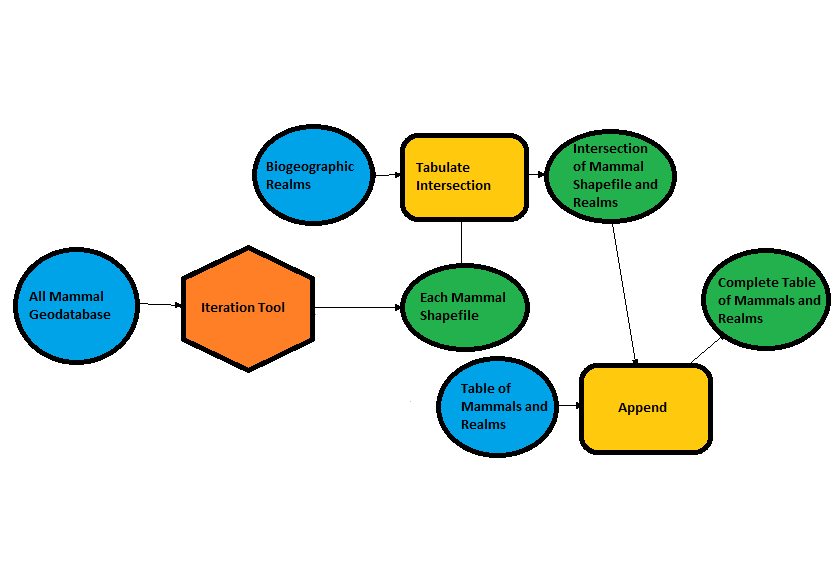


Figure S2: Species discovery curves for select biogeographic realms in which there was enough data for estimates to be made. Mammal species discovered per 5 year intervals on a log scale in each realm. The dashed blue lines represent the confidence interval around the estimated number of species in each five-year interval (S­_iest_).


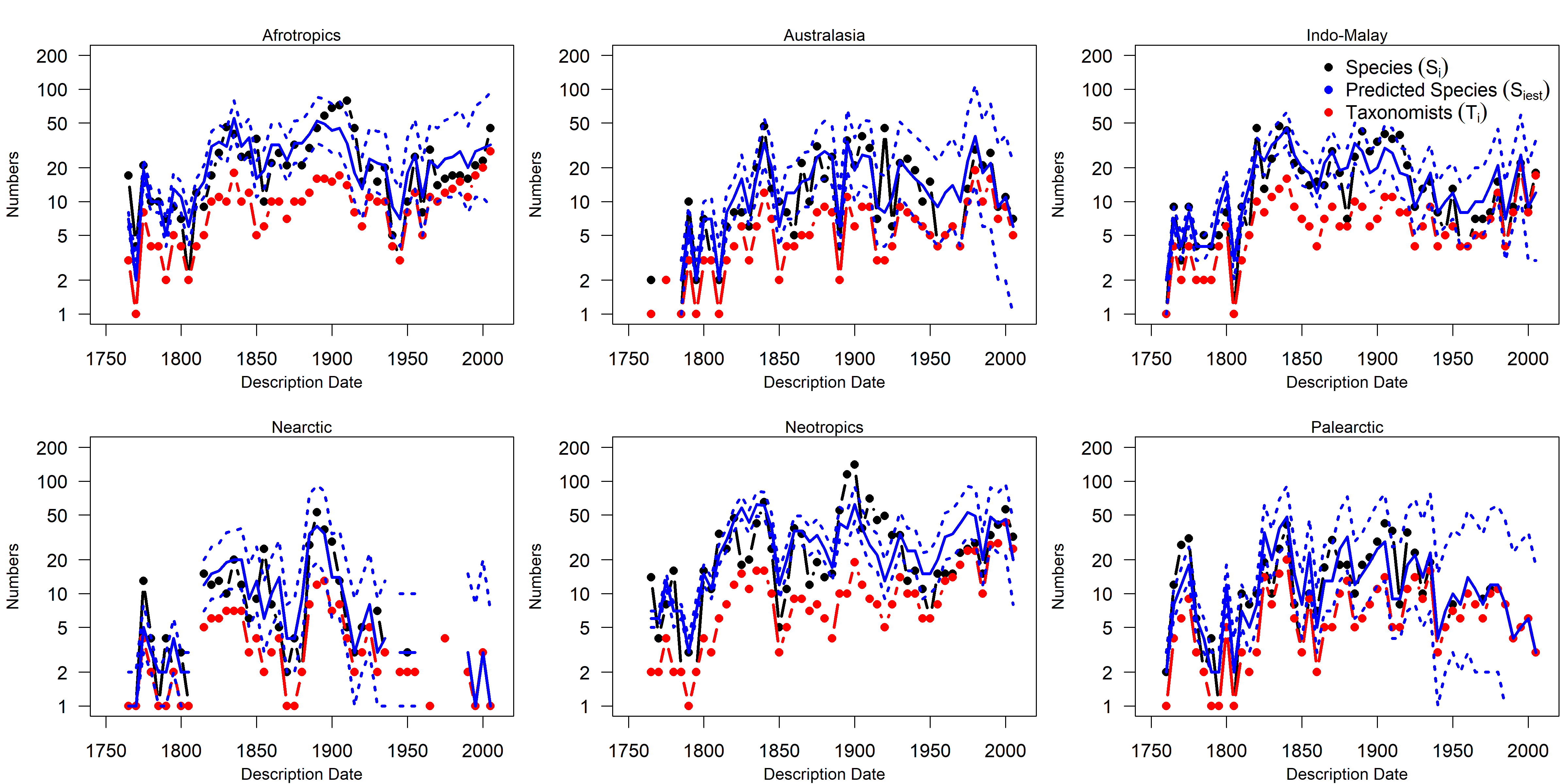

Supplement: Supplementary file 1 [file ECE3-8-3628-s001.docx]
